# An innovative organoid model to screen for bacteria and their metabolites that protect against inflammation-induced colonic barrier disruption

**DOI:** 10.1128/msphere.00926-25

**Published:** 2026-07-31

**Authors:** Nicole Cady, Sophia R. Meyer, Kwi Kim, Bernard C. Lo, Ngai So, Grace Y. Chen, Stephan P. Rosshart, Yatrik M. Shah, Gabriel Núñez, Jonathan Z. Sexton, Thomas M. Schmidt

**Affiliations:** 1 Department of Microbiology and Immunology, University of Michigan 1259, Ann Arbor, Michigan, USA; 2 Department of Medicinal Chemistry, University of Michigan 1259, Ann Arbor, Michigan, USA; 3 Department of Internal Medicine, University of Michigan 1259, Ann Arbor, Michigan, USA; 4 Department of Pathology, University of Michigan 1259, Ann Arbor, Michigan, USA; 5 Rogel Cancer Center, University of Michigan 1259, Ann Arbor, USA; 6 Department of Microbiome Research, University Hospital Erlangen, Friedrich-Alexander-Universität Erlangen-Nürnberg (FAU) 236190, Erlangen, Germany; 7 Department of Medicine 1, University Hospital Erlangen, Friedrich-Alexander-Universität Erlangen-Nürnberg (FAU) 551451 , Erlangen, Germany; 8 Center for Infectious Disease Education and Research (CIDER), Osaka University 13013, Suita, Japan; 9 Department of Ecology & Evolutionary Biology, University of Michigan 1259, Ann Arbor, Michigan, USA; University of California Davis, Davis, California, USA

**Keywords:** primary human colonoid, physiological hypoxia, gastrointestinal permeability, inflammation, *Bifidobacterium*, indole-3-lactic acid

## Abstract

Organoids provide a useful model system to study interactions between gut microbes and the activity of host tissues, including gastrointestinal (GI) barrier function. However, most organoid models have limited capacity to mimic the physiological hypoxia experienced by the colonic epithelium *in vivo*. While oxygen is required to support respiration in the host cells, many gut microbes are anaerobes that require anoxic conditions to grow. Using an innovative anaerobe co-culture system that maintains constant oxygen concentrations on the basolateral side of colonic epithelial monolayers and anoxic conditions on the luminal side, we demonstrate that colonic monolayers derived from a primary human cell line behaved more like *in vivo* epithelia when cultivated in the co-culture system than when cultured under ambient oxygen concentrations. We then identified interactions between the colonic epithelium and a strain of *Bifidobacterium adolescentis*—an oxygen-sensitive species that is prevalent and abundant in the adult gut microbiome. Co-culturing colonic monolayers with *Bifidobacterium adolescentis* U269-1 in the anoxic apical chamber mitigated loss of the barrier function in monolayers exposed to inflammatory cytokines. We identified indole lactic acid (ILA) as one metabolite from *B. adolescentis* that provides dose-dependent protection against inflammatory cytokines. Oral administration of ILA was well tolerated in mice and provided protection against immunotherapy-induced (anti-CTLA4) colitis in female, but not male mice. Together, we demonstrated the utility of the co-culture system for screening microbes and their metabolites for interactions with the colonic epithelium, as well as advancing our understanding of interactions between bifidobacteria and host barrier function.

**IMPORTANCE:**

The present study yielded strong evidence for the beneficial effects of *B. adolescentis* on the maintenance of colonic barrier function in an asymmetric oxygen system that mimics the colonic environment. The bifidobacterial metabolite indole lactic acid was implicated in this protection both *in vitro* and in a mouse model. We also refined and scaled the co-culture system to a 96-well plate format to provide the capacity for higher-throughput screening of microbes and microbial metabolites under physiologically relevant conditions.

## INTRODUCTION

Bifidobacteria are prominent members of the healthy human gut microbiome. They dominate the infant microbiome and remain as common members of the gut microbiome through adolescence into adulthood, though the species profile changes with age. The most abundant *Bifidobacterium* species in infants *B. longum subspecies infantis*, *B. bifidum*, and *B. breve* (1, 2) are used regularly as probiotics and have received more attention in research than those species abundant in the adult microbiome, in part because they are more tolerant of oxygen and easier to culture in the laboratory (3).

A meta-analysis of independently published metagenomes consistently identified three different *Bifidobacterium* species—B. *adolescentis, B. angulatum,* and *B. catenulatum*—as being associated with healthy adults (4, 5). These bifidobacteria help maintain barrier function including through modulation of tight junction complexes (6, 7) and reduction of inflammation in the GI epithelium (8–10). There is a growing body of evidence suggesting that several bifidobacterial metabolites, including ILA, promote intestinal barrier function (11–13). Indole-3-lactic acid (ILA) is one such metabolite produced by both human-associated *Bifidobacterium* (14) as a byproduct of microbial nitrogen assimilation during tryptophan catabolism (15). To date, preliminary studies have been performed investigating the role of ILA in the gut (9), the immune system (8), cell and stem cell programming (16, 17), and neuroinflammation (9). Further study of the relationship between microbial production of ILA and its role in host-microbe interactions affecting gastrointestinal barrier function is needed.

To determine if *B. adolescentis* 269-1, a strain we isolated from a healthy adult, enhances colonic barrier function, we refined an *ex vivo* organoid model that provides the necessary anoxic conditions for growth of *B. adolescentis* while simultaneously providing oxygen for respiration of the epithelial cells.

Advances in the cultivation of human organoids have offered numerous opportunities to investigate host-microbiome interactions (18). For intestinal organoids, several platforms use microfluidics to create a “gut-on-a-chip” format (19). Those platforms incorporate peristalsis-like mechanical strain and fluid flow to promote the formation of three-dimensional structures in the epithelium while maintaining stable oxygen gradients, providing more precise modeling of physiological processes and disease states (20, 21). While “gut-on-a-chip” systems offer considerable flexibility and potential, the cost of the system is prohibitive for many researchers. One cost-effective alternative that also maintains physiological oxygen environments characteristic of the human colon is the anaerobe co-culture system (22).

We refined the anaerobe co-culture system to enhance its functionality and expand its applicability. Gentle shaking was added to disrupt gradients of dissolved gases and metabolites that form in static systems. A new medium was developed to maintain a circumneutral pH throughout the fermentative growth of anaerobes in the luminal chamber because changes in pH have physiological effects on the host environment and host cells (23, 24). An assay to identify microbes and metabolites that confer resistance to a challenge with inflammatory cytokines was developed, and the platform was extended to a 96-well format to facilitate higher throughput screening.

The suitability of this system that we refer to as the asymmetrical oxygen system (AOS) was tested with a strain of *B. adolescentis*. In addition to measuring barrier function in response to inflammatory cytokines, we assessed the bifidobacterium metabolite ILA on barrier function in the *ex vivo* system and *in vitro* in a mouse model of colitis.

## RESULTS

### Establishing asymmetric atmospheres to support hypoxic metabolism of colonic epithelia and growth of anaerobic microbes

**Fig 1:**
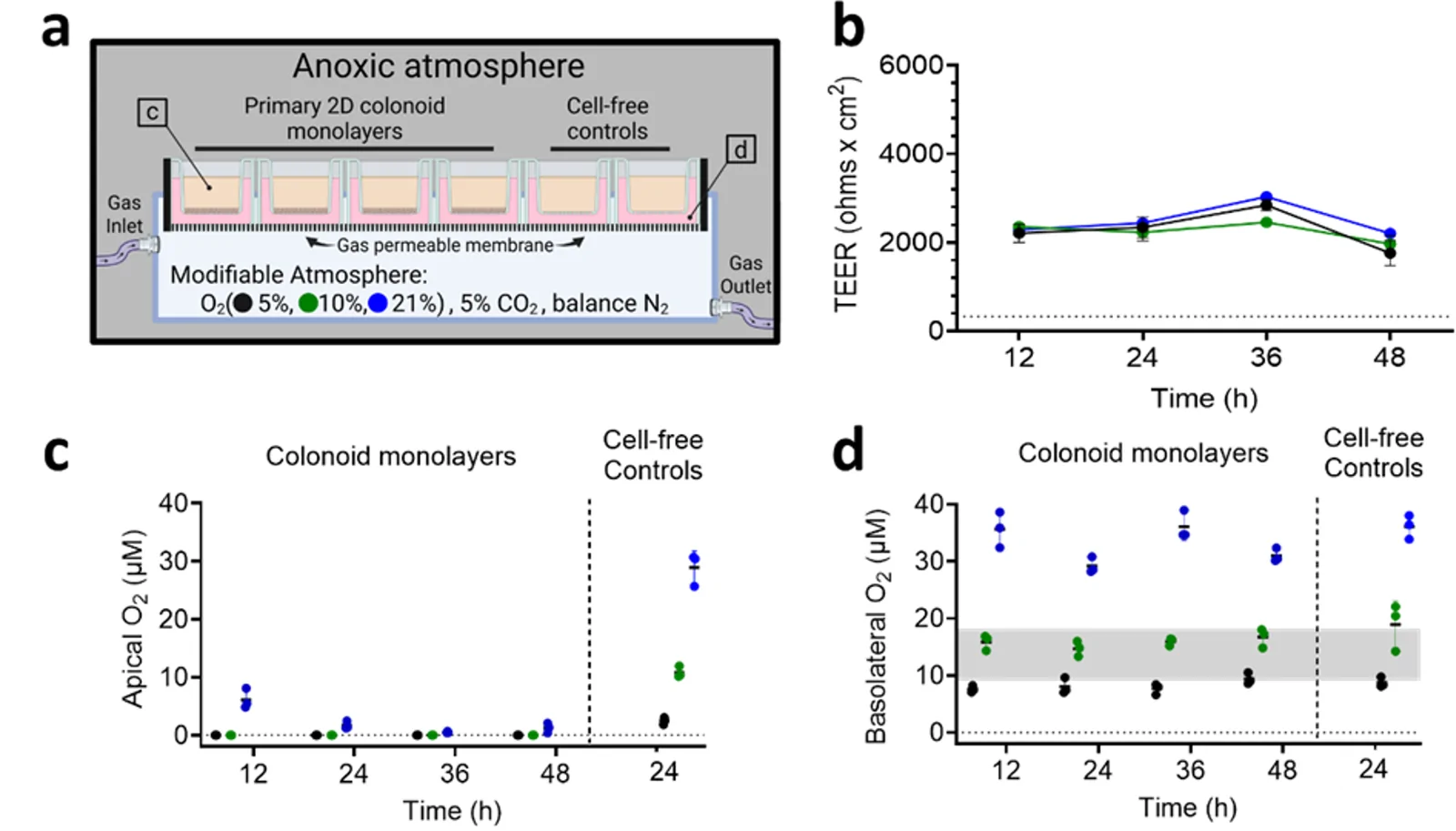
A coculture system supports the simultaneous cultivation of colonic epithelial cells and strict anaerobes. (**a**) Schematic of the coculture system in an anaerobic chamber. (**b**) Mean transepithelial electrical resistance (TEER) of colonic monolayers (*n* = 2) maintained for 48 h under asymmetric atmospheres. Dashed line in panel **b** indicates threshold for confluence (330 ohms · cm^2^) (21). (**c**) Apical and (**d**) basolateral oxygen concentrations in transwells containing a human-derived, primary colonic monolayer; cell-free measurements were conducted in sterile transwells. The gray range indicates *in vivo* oxygen concentrations in colonic epithelia of healthy humans (25). *n* = 3 transwells per group for all oxygen measurements. Error bars represent ± SD.

Colonic monolayers were established in cell culture inserts (transwells) and transferred to a receiver plate with a gas-permeable bottom when confluent (TEER ≥ 330 ohms · cm^2^). This plate, including the transwells, was then transferred to an airtight coculture chamber within a humidified anaerobic chamber (Fig. 1a). Gases, including O_22_ at the selected concentration, circulate through the bottom of the coculture chamber, diffuse through the gas-permeable membrane, and into the basolateral medium to maintain respiration in the colonic epithelium. Concentrations of 5%, 10%, and 20% oxygen (vol/vol) all provided sufficient oxygen for monolayers to maintain TEER over a 48-h period (Fig. 1b). Gas mixtures containing either 5% or 10% oxygen yielded undetectable oxygen levels in the apical chamber for 48 h when a colonic monolayer was present (Fig. 1c). Oxygen was detectable in the apical chamber when 20% oxygen was circulated through the coculture chamber (Fig. 1c). Oxygen concentrations in cell-free control transwells increased proportionally to the respective oxygen circulating in the co-culture system (Fig. 1c).

A circulating gas mix of 10% oxygen (vol/vol) provided an average oxygen concentration of 15.82 ± 1.13 µM in the basolateral chamber (Fig. 1d), which is within the range of oxygen concentrations measured at the crypt-lumen interface of the colon in humans (gray bar; 9.1–18.2 µM). We observed similar oxygen concentrations in the 96-well transwell AOS (Fig. S1). A 10% oxygen mix was selected for all subsequent experiments, and we refer to these conditions—hypoxic basolateral chamber and anoxic apical chamber—as “asymmetric atmospheres.”

To cultivate microbes in direct association with a colonic monolayer without impeding the growth and metabolism of either cell population, we developed a co-culture medium (CCM; Table S1) that supported bacterial growth without disrupting the monolayer. Several mammalian-derived bacteria from multiple phyla were evaluated for growth in CCM. All grew robustly in this medium and resulted in minor changes in the pH of the medium (Table S2).

### Responses of colonic monolayers to asymmetrical atmospheres

**Fig 2:**
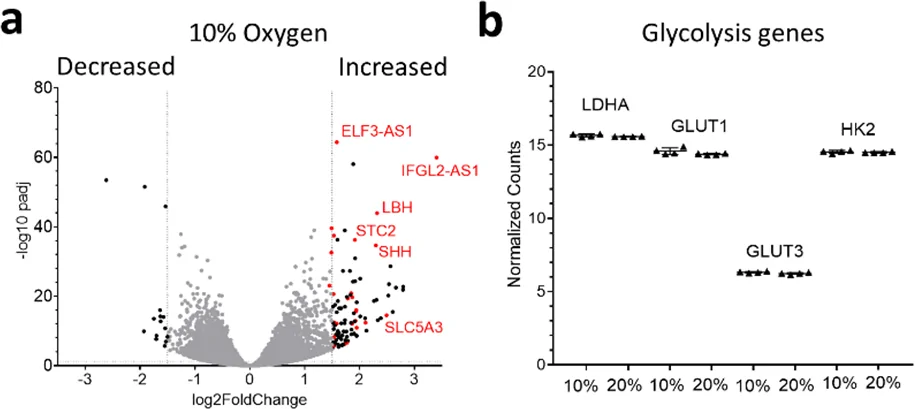
2D colonoids respond to physiological oxygen conditions. (**a**) Differentially expressed genes (DEGs) from organoids maintained in asymmetrical atmospheres or 5% CO_2_ incubator with ambient oxygen for 24 h. Volcano plot is based on Benjamini-Hochberg adjusted Wald test *P*-value. Cutoffs were set at FDR < 0.05 and linear fold change > |1.5|. Red points indicate hypoxia-responsive genes identified in the literature (Table S3). (**b**) Read counts of selected glycolysis genes under asymmetric 10% oxygen or atmospheric oxygen conditions showing lack of induction of genes associated with hypoxia-induced cell death (25). Each point represents gene counts from a transwell normalized to sequencing depth using regularized log transformation (DESeq2), *n* = 4 transwells. Bars represent mean, error bars ± SD.

To determine whether the asymmetrical atmospheres induce cellular hypoxia responses without evidence of detrimental cell stress, we cultured monolayers under asymmetric atmospheres with 10% (vol/vol) oxygen gas as described above, or in a standard 5% CO_2_ incubator exposed to ambient (~20%) oxygen. RNA-Seq identified a total of 2,423 differentially expressed genes (linear fold change > |1.5|, adjusted *P*-value < 0.05) (Fig. 2a). In the top 10% of significantly upregulated genes (140 genes total; Table S3), 40 genes have been previously identified as hypoxia-related (Fig. 2a, red). Investigation of well-characterized intracellular glycolysis genes (*LDHA, GLUT1, GLUT3, HK2*) revealed a lack of differential expression that has been observed in severe hypoxia (Fig. 2b). Together, this is consistent with a model of mild physiological hypoxia, and we conclude that the monolayers respond to the reduced oxygen conditions when compared with ambient (~20%) oxygen exposure.

### Co-culture of *B. adolescentis* with colonic monolayers in a model of barrier disruption

**Fig 3:**
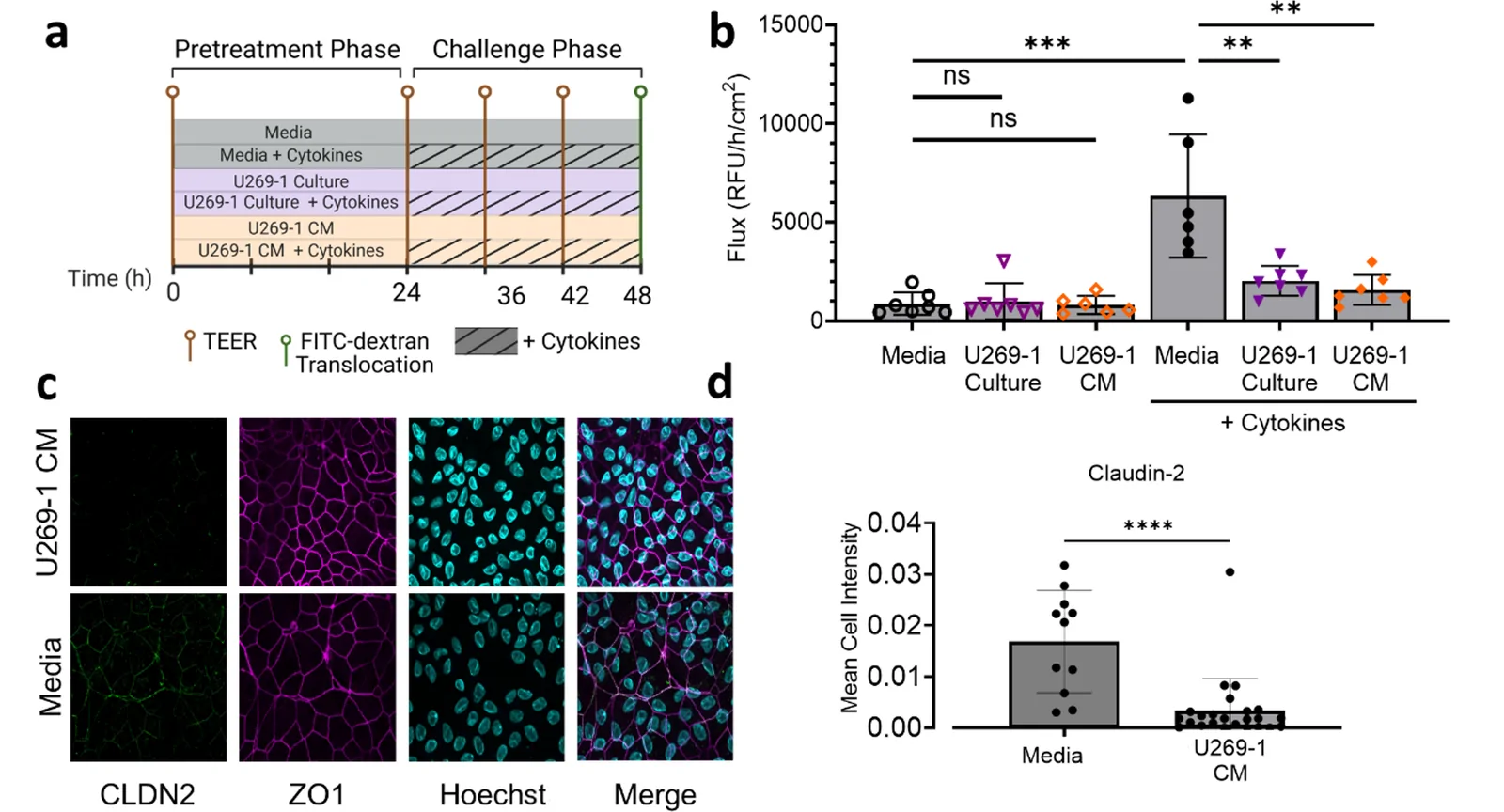
*Bifidobacterium adolescentis* metabolites reduce inflammatory cytokine-induced intestinal barrier disruption in a primary human colonoid model. (**a**) Experimental design. Transwells were transferred into asymmetrical atmospheres at time 0. Pretreatments were maintained on the transwells through the end of the experiment. Inflammatory cytokines TNFα and IFNγ (12.5 ng/mL each) were added at 24 h and maintained until 48 h. Barrier permeability was assessed periodically by TEER and at 48 h by FITC-dextran translocation assay. (**b**) FITC-dextran translocation is not increased by actively growing culture of *B. adolescentis* strain U269-1 (U269-1 culture) or cell-free conditioned media from *B. adolescentis* strain U269-1 (U269-1 CM). Both treatments are protective against inflammatory cytokine-induced increase in permeability. (**c**) Representative immunofluorescence images of monolayers maintained under asymmetrical atmospheres for 48 h with conditioned media from *B. adolescentis* strain 269-1 (269-1 CM) or control (Media) showing claudin-2 (green), ZO-1 (magenta), and nuclei (cyan). (**d**) Quantified mean cell intensity of claudin-2 from organoids in panel **c**. Eleven technical replicates were analyzed from *n* = 1 transwells (media) and 12 technical replicates from each *n* = 2 transwells (269-1 CM). (****P < 0.0001, ***P < 0.001, **P < 0.01; Mann-Whitney *U* test). Bars represent mean, error bars ± SD.

To determine if bifidobacteria can modulate a health-relevant function of the intestinal epithelium, we developed an inflammatory disruption model in which monolayers were first exposed to bacteria or bacterial products (“pretreatment phase”), followed by the addition of inflammatory cytokines (“challenge phase”) (Fig. 3a). Monolayers were exposed to a combination of TNFα and IFNγ in the challenge phase to simulate host GI inflammation, then TEER was monitored for 24 h before assaying total barrier permeability with a 4kD fluorescein isothiocyanate (FITC) labeled dextran (Fig. 3a). Cytokine-treated monolayers displayed increased permeability to the 4kD dextran compared with medium controls (Fig. 3b), indicative of a disrupted barrier.

To test whether *B. adolescentis* could prevent the cytokine-induced barrier disruption, monolayers were co-cultured with *B. adolescentis* strain U269-1 during the pretreatment phase. There was no change in permeability relative to the medium control during the pretreatment phase, demonstrating that monolayers tolerate the co-culture (Fig. 3b). Notably, these monolayers were resistant to barrier disruption when exposed to inflammatory cytokines (Fig. 3b). This protection was also induced by pretreatment with cell-free culture media in which U269-1 had grown. The protective effect of the culture supernatant was reproduced in the 96-well adaptation of the AOS system (Fig. S2).

We next asked if the mechanism of resistance was due to enhanced production of tight junction proteins. Monolayers were treated with either fresh CCM or 269-1 conditioned medium alone before fixation and imaging (Fig. 3c). There was no change in tight junction protein ZO-1 intensity; however, the intensity of “leaky” junction protein claudin-2 was reduced in monolayers treated with *B. adolescentis*conditioned medium (Fig. 3d).

### Treatment of colonic monolayers with bifidobacteria metabolite ILA in a model of barrier disruption

**Fig 4:**
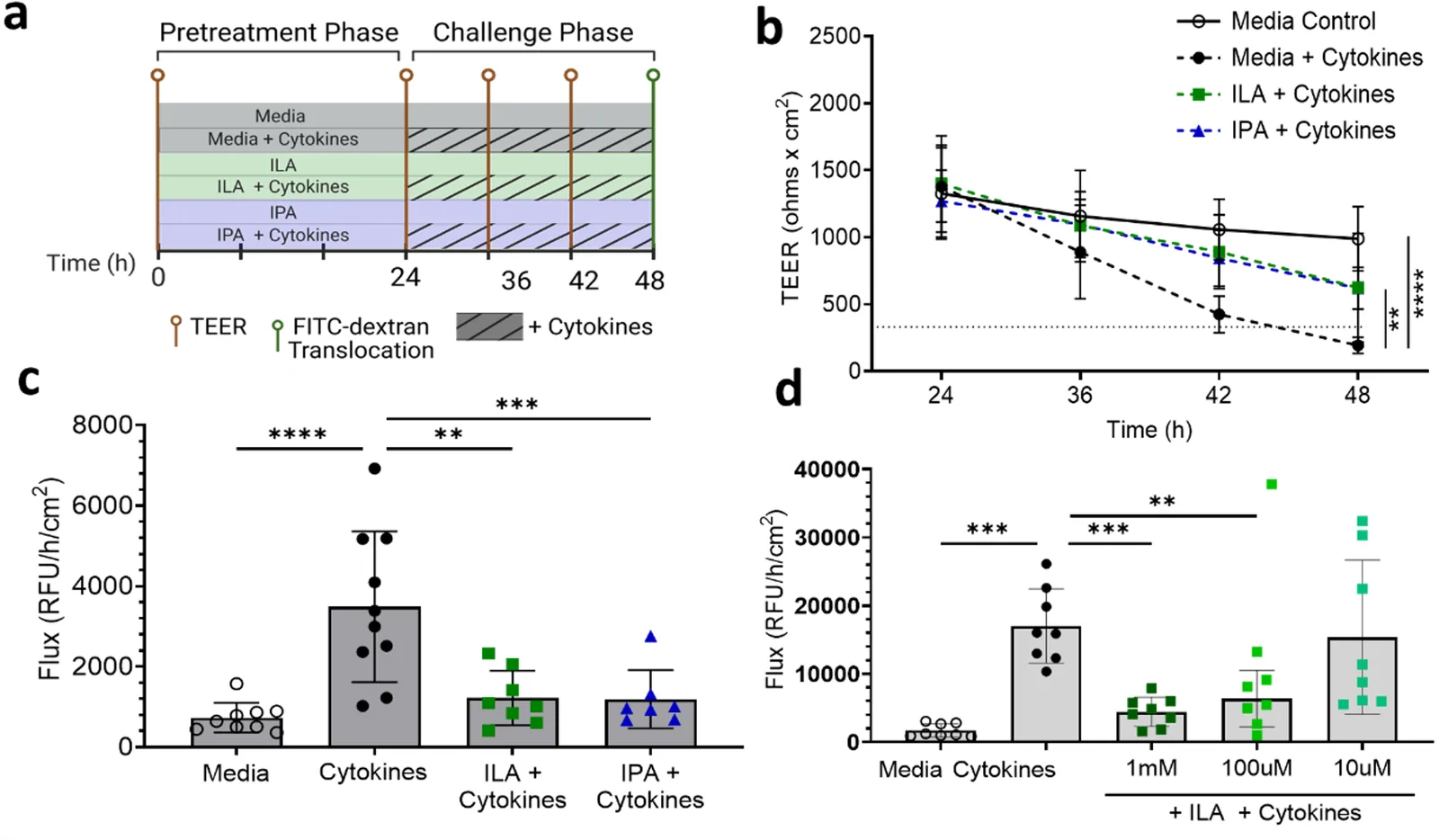
ILA protection against inflammatory cytokine-induced intestinal barrier disruption is dose-dependent. (**a**) Experimental design. Transwells were transferred into asymmetrical atmospheres at time 0. Prereatments were maintained on the transwells through the end of the experiment. (**b**) Mean TEER values (Ω · cm^2^) over 48 h under asymmetrical atmospheres. Dashed line indicates confluence (330 Ω · cm^2^) (18). Significance values correspond to percentage change in TEER after addition of inflammatory cytokines (24 to 48 h). (**c and d**) FITC-dextran translocation at 24 h post addition of cytokines. Flux refers to relative fluorescence units (RFU)/time/medium volume/membrane area. Each point represents data from one colonoid monolayer. Data pooled from three independent experiments in panel **c** for all conditions except IPA + cytokines, in which two independent experiments were performed. Data pooled from two independent experiments in panel **d**. Outlier shown but excluded from analysis in panel c, 100 μM group based on Tukey’s fences 1.5*IQR rule. Bars represent mean, error bars ± SD (****P < 0.0001, ***P < 0.001, **P < 0.01; Mann-Whitney *U* test).

ILA is one metabolite produced by *B. adolescentis* U269-1 (Fig. S3) that has the potential to influence the host epithelium. Using a similar experimental design to the co-culture experiment, monolayers were pretreated with ILA (1 mM) or the chemically related bacterial metabolite indole-3-propionic acid (IPA) for 24 h before challenge with inflammatory cytokines (Fig. 4a). Both compounds protected against barrier disruption, as measured by TEER (Fig. 4b) as well as FITC-dextran permeability (Fig. 4c). Additional assays showed that the effect of ILA is dose-dependent: 100 µM ILA was sufficient to provide protection, but 10 µM was not (Fig. 4d). This result was repeated in the high-throughput 96-well system (Fig. S4a). Additionally, we demonstrated that the ability of both ILA and U269-1 CM to protect against cytokine-induced barrier disruption is lost in the presence of aryl hydrocarbon receptor (AhR) inhibitor CH223191 (Fig. S4b). From this, we conclude that ILA from *B. adolescentis* can provide some protective function by maintaining the barrier function of the colonic epithelium via its role as an AhR agonist.

### Treatment of mice with ILA in a model of cancer immunotherapy-associated colitis

**Fig 5:**
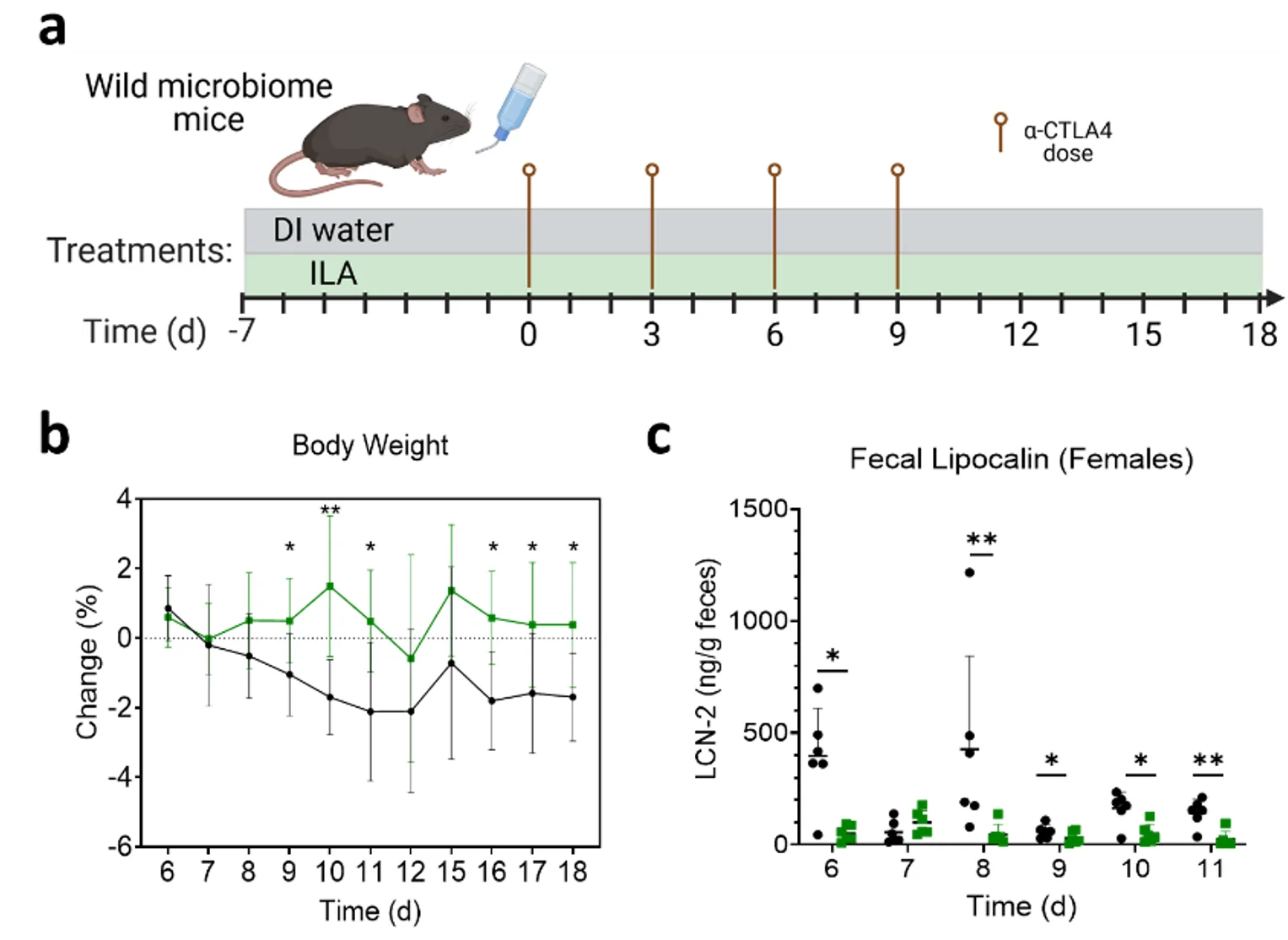
ILA protects against colitis in a wild microbiome mouse model. (**a**) Experimental design: 1 mM ILA in DI water or DI water alone was administered *ad libitum* for the duration of the experiment. Then, 100 μg doses of α-CTLA4 antibody were administered IP every 3 days. (**b**) Change in body weight after administration of anti-CTLA4. Each point represents the mean of 5–6 mice, error bars ± SD. (**c**) Fecal lipocalin levels after administration of anti-CTLA4. Each point represents one mouse, error bars + SD (****P < 0.0001, ***P < 0.001, **P < 0.01; Mann-Whitney *U* test).

To evaluate the effect of ILA on a microbiome-dependent model of colitis, we used mice colonized with a gut microbiota originally derived from wild-caught mice, referred to as wild mouse microbiome-reconstituted (WildR) mice (26). WildR mice are susceptible to colitis when treated with α-CTLA4 antibody (27). Then, 1 mM ILA or DI water control was administered via drinking water for 7 days prior to induction of colitis (Fig. 5a). Female control mice showed a moderate reduction in body weight starting on day 8, (Fig. 5b) as well as GI inflammation as measured by fecal lipocalin-2 (Fig. 5c), while female mice consuming ILA were protected from both loss of body weight and GI inflammation. No protection was seen in male WildR mice consuming ILA compared with controls (Fig. S5).

## DISCUSSION

To determine if a cultivar of *B. adolescentis*—an oxygen-sensitive species common to the adult microbiome—enhances resistance of the colonic epithelium to disruption by inflammatory cytokines, we refined an *ex vivo* co-culture system to better mimic the environmental conditions of the human colon. *B. adolescentis* is of particular interest because of its prevalence in healthy adults, along with its ability to increase in abundance in individuals who supplement their diet with resistant potato starch (28). Resistant potato starch supplementation is a simple and cost-effective dietary intervention that, if paired with the actions of beneficial bacterium, may help address intestinal inflammation. Cultivars from multiple species of *Bifidobacterium* improve intestinal barrier function in cell and rodent models (29–32), but human cell models typically use transformed cell lines, which mimic the polarization of the intestinal epithelium but display altered signaling and functions compared with healthy tissue (33). Additionally, most cell culture models are performed under ambient oxygen atmospheres. These approaches are not suitable for investigation of oxygen-sensitive strains of bacteria that are common in the adult microbiome. To enable discovery of beneficial anaerobic microbes and their metabolites that influence barrier function under physiological conditions, we modified a recently described system to coculture anaerobic microbes with a colonic monolayer (22). We added gentle shaking at regular intervals to diffuse chemical and oxygen gradients that form in static cultures (34) and to provide low levels of shear stress (35) that influence gene expression in epithelial cells (36). To avoid inconsistencies between experiments due to cell senescence (25, 37, 38), we used a primary human organoid-derived cell line that was passaged in a narrow, predefined range (16–20 total passages).

A 10% oxygen gas mixture in the AOS was chosen to reproduce oxygen concentrations reported in the colonic epithelium (39). Under these conditions, there was no measurable oxygen in the apical chamber, making it suitable for the growth of anaerobic microbes. To assess the transcriptional status of the epithelial cells under these conditions, RNA-Seq data from monolayers incubated under the asymmetric oxygen concentrations or room air supplemented with 5% CO_2_ atmosphere were compared. Glycolysis genes, which are upregulated under anoxic atmospheres or those containing 1% O_2_ (40), were not upregulated in our model system, suggesting that the monolayers received sufficient oxygen for respiration. As expected, several hypoxia-associated genes were upregulated, suggesting that physiologically relevant oxygen concentrations were achieved.

To facilitate bacterial culture in the asymmetric culture plate, we developed a co-culture medium (CCM) that both supports a functional epithelial monolayer and accommodates growth of phylogenetically diverse anaerobic bacteria from the human gut (Table S2). The medium is buffered at a pH of 6.5, corresponding to average conditions in the proximal colon (41, 42).

Using the AOS with CCM in the apical chamber, we began by asking how the intestinal epithelium responds to the presence of a growing population of *B. adolescentis* U269-1. Using an inflammatory cytokine-mediated barrier disruption assay modeled after previous studies (43, 44), monolayers were exposed to a growing culture of strain U269-1 for 24 h before addition of inflammatory cytokines. Co-culture of monolayers with *B. adolescentis* U269-1 significantly reduced small molecule translocation across the monolayer, as measured by FITC dextran. Similar protection from inflammatory cytokine disruption of the monolayer was seen when monolayers were exposed to filtered medium from a *B. adolescentis* U269-1 culture, and so we conclude that the mechanism of protection is likely metabolite-mediated rather than requiring direct contact between the bacterium and the monolayer. Heat-inactivated bifidobacteria have previously shown protective effects in both colitis (45) and organoid models (46), potentially implicating cellular compounds such as surface-associated or secreted extracellular polysaccharides as cell components that provide additional protection.

Given previous observations of the effects of bifidobacteria on tight junction proteins (30), we used immunofluorescence to visualize intensity and localization of tight junction proteins in monolayers treated with CCM or U269-1 conditioned medium. Claudin-2 is a “leaky” or pore-forming tight junction protein with a pathogenic role in colitis (47), and its expression was decreased in monolayers exposed to strain U269-1 conditioned medium. Claudin-2 expression is repressed by agonism of the AhR (48, 49), so we took a closer look at ILA—a known AhR agonist produced by some bifidobacteria (14).

Multiple studies in both animal and cell culture models have identified ILA as a metabolite of the gut microbiota with the potential to modulate barrier function and GI inflammation (9, 50, 51). Our results generated under physiologically relevant conditions support these conclusions: chemically pure ILA provided dose-dependent protection against barrier disruption induced by inflammatory cytokines. This was further validated by the inability of ILA or U269-1 CM to provide protection in the presence of AhR inhibitor CH223191 (Fig. S5). However, given the concentrations of ILA produced by strain U269-1 (~15 µM) and the lack of protection observed from treatment with 10 μM ILA, ILA is likely only one of the bioactive metabolites contributing to the beneficial effects of U269-1. Interestingly, ILA provided sufficient protection to maintain epithelial barrier function as measured by both TEER and small molecule translocation, while *B. adolescentis* U269-1 CM was only protective against small molecule permeability at 24 h post-addition of cytokines (Fig. S2). This may indicate that providing a high dose of ILA has a different effect on overall barrier physiology and/or tight junction structure than lower dose from U269-1. This, along with other potentially bioactive molecules, may influence the epithelial barrier differently, still providing important functional protection with physiological implications for GI inflammation.

Finally, to extend the *in vitro* demonstrations of the beneficial effects of ILA on colonic barrier functions to an *in vivo* model, we employed a cancer immunotherapy-induced colitis model in mice. Unlike conventional laboratory mice, these mice have a wild mouse microbiome, which makes them susceptible to anti-CTLA4 colitis (27). We chose this model because the GI inflammation is driven in part by the inflammatory cytokine IFNγ + CD4 cells + T cells (27), complementary to our barrier disruption model in the organoid system. ILA consumption via drinking water was protective against both reduction in body weight (Fig. 5b) and GI inflammation—but only in female mice (Fig. 5c). Interestingly, no protection was seen in male wild mice consuming ILA (Fig. S2). Recent studies investigating the role of ILA in Alzheimer’s disease also showed that only females responded (52).

We confirmed that the AOS provided similar oxygen levels in both 24- and 96-well formats (Fig. S1), and that functional observations in the inflammatory cytokine-induced disruption model were consistent in both formats (Fig. S2 and S4). This demonstrates the high translational potential of the AOS, similar to other high-throughput models that predict clinical trial results for drug toxicity (53, 54) and chemosensitivity (55, 56).

With the medium formulations we used, the intact monolayer is viable for just a few days. By modifying the growth factors present in the cell culture media, colonic epithelia have now been maintained in a transwell model for up to 30 days (57). This extended lifetime of a viable monolayer makes it suitable for broader drug screening and for chronic interaction studies.

The results presented describe the beneficial effects that *B. adolescentis* in maintaining intestinal barrier function in the presence of inflammatory cytokines. They identify ILA as one metabolite that, via AhR agonism, helps reinforce the colon epithelium. The AOS demonstrates an experimental framework for investigating host-microbe interactions in the gut, particularly with oxygen sensitive anaerobes like adult-type bifidobacteria, ILA and other microbial metabolites. Moreover, they demonstrate the potential for organoid models in drug screening, especially in the evolving space of live biotherapeutic products. In complement, studies from our lab show that consumption with resistant starch from potatoes can increase the relative abundance of *B. adolescentis* in healthy participants. Together, this provides a mechanism for how simple dietary intervention may have highly beneficial effects on GI integrity, and justification for further study in populations that may benefit from a reinforced GI barrier.

## MATERIALS AND METHODS

### Organoid culture

The primary human colonic epithelial organoid cell line (Col81) was obtained from cryopreservation three generations post-derivation from the University of Michigan Human Organoid Core (https://www.umichorganoid.org/: RRID:SCR_027333). This cell line was derived from a biopsy of normal tissue from the ascending colon, expanded and cryopreserved, as previously described (https://www.umichorganoid.org/protocols) (58). We used similar methods to propagate 3D organoid cultures and then seed them into 2D transwell monolayers. Briefly, cryopreserved colonoid fragments were seeded into Cultrex UltiMatrix Reduced Growth Factor Basement Membrane Extract (Biotechne, cat# BME001) to support 3D growth for 7–14 days in a humidified 37°C incubator supplied with 5% CO_2_ (balance room air). Organoids were then dissociated by gentle passage through a 200-μL pipette tip while on ice and resuspended in fresh Cultrex Gel every 7 days for up to a total of 35 days (four total passages). 3D organoids were maintained in human colonoid medium (HCM; adapted from https://www.umichorganoid.org/protocols), with supplements A8301 (500nM; Tocris-Fisher Cat#29-391-0), SB202190 (10uM; Sigma, Cat# S7067), Y27632 (10uM; Tocris, Cat#1254).

### Colonic monolayers

Colonic monolayers were seeded in transwells with 300K (24-well format; Corning, cat# 3470) or 150K (96-well format; Corning, cat# 7369) cells per well in HCM for 20 h in a 5% CO_2_ incubator at 37°C with ambient oxygen. Cells were then transferred to an incubator with similar conditions but reduced (10%) oxygen. At this time, media were changed from HCM to two-dimensional medium (2DM). 2DM contains DMEM/F12, supplemented with N-2 (1×), B-27 minus VitA (1×), Glutamax (2 mM), HEPES (1 0mM), Noggin (50 ng/mL), Y-27632 (2.5 µM; added for the first 48 h only), EGF (50 ng/mL), gastrin (10 nM), and N-acteylcystine (1 mM). Upon establishment of confluence (TEER > 330 ohms · cm^2^) (59), apical 2DM was replaced with CCM (Table S1).

### Asymmetric atmospheres

Transwells were transferred into the Anaerobic Co-Culture system (Coy Laboratories, Cat# 8704000) including a gas permeable plate (Zell Kontakt, catalog #3231, Germany). A gas mixture containing oxygen (5%, 10%, or room air) and 5% CO_2_ was circulated beneath the gas permeable plate through the coculture chamber, while the apical chamber was exposed to the anaerobic atmosphere of a Coy chamber (Coy Laboratories) at 37°C. This coculture chamber was placed atop a shaker programmed to gently agitate the system for 5 min every hour to help disrupt any gradients that might form.

### RNA sequencing

RNA was isolated using RNeasy Micro Kit (Qiagen cat no. 74004). Sequencing was performed by the UM Advanced Genomics Core, with libraries constructed and subjected to 150 paired-end cycles on the NovaSeq-6000 platform (Illumina). Data were pre-filtered to remove genes with 0 counts in all samples. Differential gene expression analysis was performed using DESeq2 (60), using a negative binomial generalized linear model (thresholds: linear fold change > 1.5 or < −1.5, Benjamini-Hochberg FDR (*P*adj < 0.05). Plots were generated using variations of DESeq2 plotting functions and other packages with R version 4.3.2. Annotation data from ENSEMBL 113 were used, and genes were additionally annotated with Entrez GeneIDs and text descriptions. Gene counts were provided after using DESeq2 regularized log transformation to normalize to sequencing depth.

### High-content imaging, processing, and cell segmentation

Twenty-four-well transwells were fixed in ice-cold 50% methanol/50% acetone solution, blocked and permeabilized with 3% BSA and 0.3% Triton for 1 h on a rotator, then stained ZO-1 AF596 (ThermoFisher Cat #339194; 1:200) and Claudin-2 MH44 (Invitrogen Cat# 51-6100; 1:100) overnight at 4°C protected from light. Cells were washed and stained with secondary antibody (Invitrogen Cat# A11008; 1:1000) and PhenoVue Hoechst 33,342 (Revvity Cat# CP71; 1:2000) for an hour at room temperature prior to imaging. Membranes were extracted and mounted in a 2.5 M fructose-glycerol optical clearing solution. High-content imaging was performed on the Yokogawa CellVoyager CQ1 Benchtop High-Content Analysis System using a 60× dry objective, and maximum intensity projections were acquired from five 3-µm Z-stack images. Sixteen fields of view were acquired for each well. Cell nuclei were segmented using Cellpose 3.0 from Hoechst-stained images, and individual cells were segmented using a custom Cellpose 3.0 model trained on ZO-1 images. Image, cell, and nuclear measurements were acquired using CellProfiler 4.2.5.

### Bacteria

*B. adolescentis* U269-1 was obtained from a healthy human study participant (8). Strain was purified, verified by PCR, and stored at −80°C. For metabolite analyses, cultures were centrifuged at 5,000 RCF at 4°C for 10 min. Supernatant was stored at −20°C for later use in either colorimetric analysis and/or LC-MS.

### Treatments

Cultures of *B. adolescentis* strain U269-1 were grown in YCFE media, a modified version of YCFA (61) containing only branched-chain fatty acids, 2 g/L Bacto casitone, vitamins tetrahydrofolic acid and pantetheine, and 100 mM MOPS. Cells were washed with fresh CCM and diluted to OD ~0.01 in CCM prior to application to the transwells in the apical chamber. For conditioned medium treatments, cultures were grown for 48 h, centrifuged at 5,000 × *g* to remove bacteria, then passed through a 0.22-μm filter to assure the preparation was bacterium-free before application to the apical chamber. Inflammatory challenges were performed using 12.5 ng/mL TNFα (R&D Systems, cat# 210-TA-100/CF) and 12.5 ng/mL IFNγ (R&D Systems, cat# 285-IF-100) in 2DM applied concurrently to the basolateral chamber. AhR inhibitor CH223191 (Tocris, cat# 3858) was added at a concentration of 10 µM.

### Transepithelial electrical resistance (TEER)

TEER was measured using an Epithelial Volt/Ohm Meter (EVOM) 3 (World Precision Instruments) and an STX4 EVOM electrode (World Precision Instruments, Cat#EVM-EL-03-03-01). The ohmmeter calibrated was evaluated using the 1,000 Ω electrode prior to each experiment. Reported values are adjusted to the surface area of the insert (0.33 cm^2^).

### FITC-dextran translocation assay

4kD FITC-dextran (Sigma-Aldrich, Cat# 46944-500MG-F) was reconstituted in sterile water at a concentration of 50 mg/mL and stored at 4°C protected from light. Then, 4 μL was added to the apical chamber, and transwells were maintained in total darkness for the length of the assay. Flux was calculated as relative fluorescence units per basolateral medium volume (800 or 200 μL)/time (in h)/area of the transwell membrane (0.33 or 0.143 cm²) for 24- and 96-well formats respectively.

### ILA measurements

Van Urk and Salkowski reagents were prepared, as previously published (62). Reagents were mixed 1 volume Van Urk to two volumes Salkowski reagents directly before the assay. Equal volumes of this working stock and cell-free conditioned media were allowed to incubate for 8 h in darkness at 37°C. Full wavelength scans on a UV-Vis spectrophotometer determined that ILA peaks at 626 nm. Standard curves with known concentrations of ILA were created to quantify ILA in the culture samples. Cell numbers between cultures were estimated to be similar by optical density at 600nm.

### Mice

Germ-free C57BL/6 mice were originally purchased from Jackson Laboratory and maintained at the University of Michigan Germ-Free Animal Core facility. Germ-free condition was verified weekly by both aerobic and anaerobic cultures and Gram staining of fecal pellets and cage bedding. All mice were bred and maintained under specific pathogen-free conditions.

### Fecal microbiota transfer

Germ-free mice were colonized as previously published (27). Briefly, freshly collected fecal pellets from C57BL/6 mice harboring wild microbiota (26) were homogenized in reduced PBS and passed through a 100-µM cell strainer in an anaerobic chamber. Then, 200 µL of homogenate was administered on three consecutive days by oral gavage. Mice containing the wild microbiome were then set as breeding pairs. Pups were used for all subsequent experiments.

### Indole-3-lactic acid administration and immune checkpoint inhibitor treatment

DI water containing 1mM ILA (Sigma, Cat# I5508) or DI water alone was administered *ad libitum* for 7 days. Then, 100 μg of anti-CTLA-4 antibody (Clone 9D9, InVivoGen) was administered by intraperitoneal injection every 3 days. Body weight was monitored daily.

### Lipocalin-2 measurements

Fecal pellets were collected and stored at −80°C until analysis. Pellets were thawed, homogenized in sterile PBS at a concentration of 0.1 g/mL, and centrifuged at 4,000 RPM for 10 min at 4°C. Supernatants were isolated and diluted for quantification using Mouse Lipocalin-2 DuoSet ELISA kit (R&D Systems).

## Data Availability

The transcriptome data discussed in this article have been deposited in NCBI’s Gene Expression Omnibus and are accessible through GEO Series accession number GSE337888.

## SUPPLEMENTAL MATERIAL

Fig. S1 msphere.00926-25-s0001.tif Oxygen measurements.

Fig. S2 msphere.00926-25-s0002.tif *B. adolescentis* U269-1 CM provides protection against cytokines in 96-well transwell format.

Fig. S3 msphere.00926-25-s0003.tif Quantification of ILA produced by *B. adolescentis* strain U269-1 in CCM.

Fig. S4 msphere.00926-25-s0004.tif ILA shows dose-dependent protection against cytokines in 96-well transwell format.

Fig. S5 msphere.00926-25-s0005.tif Male mice fed ILA show no protection from anti-CTLA4 colitis.

Supplemental material msphere.00926-25-s0006.docx Legends for supplemental files; Table S2.

Table S3 msphere.00926-25-s0007.xlsx RNAseq expression data.

Table S1 msphere.00926-25-s0008.xlsx Recipe for CCM.

ASM does not own the copyrights to Supplemental Material that may be linked to, or accessed through, an article. The authors have granted ASM a non-exclusive, world-wide license to publish the Supplemental Material files. Please contact the corresponding author directly for reuse.
