# Supplementary material for: An innovative organoid model to screen for bacteria and their metabolites that protect against inflammation-induced colonic barrier disruption
Source: mSphere. 2026 Jul 31;11(8):e00926-25. doi: 10.1128/msphere.00926-25 (PMC13508787; doi:10.1128/msphere.00926-25)
Supplement: Supplementary file 1 [file msphere.00926-25-s0006.docx]

**Supplemental Figures and Tables**

**Figure S1. 96-well oxygen measurements.** (a) Apical and (b) basolateral oxygen concentrations in transwells containing a human-derived, primary colonic monolayer; cell free measurements were conducted in sterile transwells. The grey range indicates *in vivo* oxygen concentrations in colonic epithelia of healthy humans (39). n=3 transwells per group for all measurements. Error bars represent ±SD.

**Figure S2. *B. adolescentis* U269-1 CM provides protection against cytokines in 96-well transwell format**. FITC-dextran translocation at 24h post addition of cytokines. Flux refers to relative fluorescence units (RFU)/time/media volume/membrane area. Each point represents data from one colonoid monolayer. Data pooled from 4 independent experiments for all conditions. Bars represent mean, error bars ±SD (**** <0.0001, *** <0.001, ** <0.01; Mann-Whitney *U* test).

**Figure S3. Quantification of ILA produced by *B. adolescentis* strain U269-1 in CCM.** (a) ILA was quantified using a Van Urk-Salkowski colorimetric assay. Each point represents measurement from an individual culture. 24 hour cultures represent conditions similar to co-culture of microbes with colonoid monolayers (U269-1 Culture) while 48 hour cultures represent conditions used to prepare conditioned media (U269-1 CM) treatments.

**Figure S4. ILA shows dose-dependent protection against cytokines in 96-well transwell format.** (a,b) FITC-dextran translocation at 24h post addition of cytokines. Flux refers to relative fluorescence units (RFU)/time/media volume/membrane area. Each point represents data from one colonoid monolayer. Bars represent mean, error bars ±SD. (**** <0.0001, *** <0.001, ** <0.01; Mann-Whitney *U* test). Panel (a) is pooled data from 2 experiments. Panel (b) presents data from one experiment.

**Figure S5. Male mice fed ILA show no protection from anti-CTLA4 colitis.** (a) Change in body weight after administration anti-CTLA4. Each point represents the mean of 5-6 mice, error bars ±SD. (b). Fecal lipocalin levels after administration anti-CTLA4. Each point represents one mouse, error bars +SD. (**** <0.0001, *** <0.001, ** <0.01; Mann-Whitney *U* test)

**Table S1. Composition of Co-Culture Medium (CCM).**

**Table S2. Growth characteristics in CCM for phylogenetically diverse bacteria isolated from the mammalian gut.**

| Species | Strain | Source | | Max OD_600nm_ | Growth rate (μ) | | Doubling time (h) | pH* |
| --- | --- | --- | --- | --- | --- | --- | --- | --- |
| *Bifidobacterium adolescentis* | 269-1 | Human | | 1.11 ± 0.03 | 0.22 ± 0.02 | | 3.21 ± 0.33 | 6.38 |
| *Bifidobacterium angulatum* | 285-2 | Human | | 0.94 ± 0.04 | 0.56 ± 0.06 | | 1.24 ± 0.13 | 6.39 |
| *Bifidobacterium longum* | 35624 | Human | | 0.54 ± 0.02 | 0.79 ± 0.26 | | 0.95 ± 0.26 | 6.46 |
| *Bifidobacterium pseudolongum* | DM57 | Mouse | | 1.02 ± 0.09 | 0.39 ± 0.21 | | 2.17 ± 0.90 | 6.02 |
| *Phocaeicolaa vulgatus^a^* | ATCC 8482^b^ | Human | | 0.94 ± 0.05 | 0.26 ± 0.08 | | 2.87 ± 0.70 | 6.41 |
| *Ruminococcus gnavus* | VPI C7-9 | Human | | 0.58 ± 0.03 | 0.14 ± 0.01 | | 5.10 ± 0.32 | 6.42 |
| *Escherichia coli* | MI_KPC_104 | Human | | 0.80 ± 0.04 | 0.19 ± 0.04 | | 3.78 ± 0.69 | 6.42 |
| ^*^Measured after 24h of growth, blank CCM measured pH 6.48 | | | | | |  |  |  |
| ^a^ Formerly *Bacteroides vulgatus* | |  | |  |  | |  |  |
| ^b^ Strain designation unavailable from ATCC | | |  |  |  | |  |  |

**Table S3. Top 10% Differentially expressed genes annotated for role in hypoxia.** Top 10% (140/1401) of upregulated differentially expressed genes (DEGs) from organoids maintained in asymmetrical atmospheres or 5% CO_2_ incubator with ambient oxygen for 24h. Cutoffs were set at FDR < 0.05 and linear fold-change > |1.5|. Annotated for published roles or connection to hypoxia.

(attached as separate Excel spreadsheet).
